# Case of Comminuted Fracture of the Skull

**Published:** 1873-11-15

**Authors:** E. Powell


### SURGICAL WARDS COOK COUNTY HOSPITAL.

CASE OF COMMINUTED FRACTURE OF THE SKULL. SERVICE OF PROF. E. POWELL. REPORTED BY D. A. K. STEELE, M.D., HOUSE SURGEON.

P. McN——, aged 45; butcher; native of Ireland; was admitted to hospital Sept. 1st. States that, six days previous to admission, he was struck on the head by a tack-hammer. Since then has had headache and pain over the frontal region. When walking has a dizzy sensation—feels as if he would fall if he did not take hold of something. On examination, find a small, round orifice over the center of frontal portion of head, discharging a small quantity of offensive pus. On introducing the probe it came in contact with the bone, denuded, roughened, and slightly depressed. General health is, and always has been, good; does not appear to have any constitutional disturbance.

Sept. 12th.—Discharge still continuing, and having the peculiar, offensive odor of dead bone. Ether was administered, and Prof. P. made a crucial incision over the wound down to the bone, and twelve or fourteen small, depressed pieces of bone were removed. The dura-mater was not injured. Patient reacted well; was kept in the horizontal posture for a few days, and evaporating lotions applied; after which a dressing of cosmoline, on lint, was applied morning and evening, after the wound had been carefully washed out with carbolated water (1 part acid carbolic to aquæ 20 parts). The wound remained open, discharging slightly, until October 31st, when it had entirely closed. During this time several small pieces of bone had appeared in the wound, and were picked out. There was no constitutional disturbance, the patient being up, walking about the ward, most of the time, seldom complaining of so much as a headache. The internal treatment consisted of small doses of quinine. Patient was discharged, recovered, November 3d.

